# Utility of the Rose Bengal Test as a Point-of-Care Test for Human Brucellosis in Endemic African Settings: A Systematic Review

**DOI:** 10.1155/2020/6586182

**Published:** 2020-09-16

**Authors:** Abel B. Ekiri, Christopher Kilonzo, Brian H. Bird, Elizabeth VanWormer, David J. Wolking, Woutrina A. Smith, Honorati Masanja, Rudovick R. Kazwala, Jonna A. K. Mazet

**Affiliations:** ^1^One Health Institute and Karen C. Drayer Wildlife Health Center, School of Veterinary Medicine, University of California, Davis, CA 95616, USA; ^2^School of Veterinary Medicine, University of Surrey, Guildford GU2 7AL, UK; ^3^School of Veterinary Medicine & Biomedical Sciences, School of Natural Resources, University of Nebraska, Lincoln, NE 68583-0905, USA; ^4^Ifakara Health Institute, Dar Es Salaam, Tanzania; ^5^College of Veterinary Medicine and Biomedical Sciences, Sokoine University of Agriculture, Morogoro, Tanzania

## Abstract

In endemic African areas, such as Tanzania, *Brucella* spp. cause human febrile illnesses, which often go unrecognized and misdiagnosed, resulting in delayed diagnosis, underdiagnosis, and underreporting. Although rapid and affordable point-of-care tests, such as the Rose Bengal test (RBT), are available, acceptance and adoption of these tests at the national level are hindered by a lack of local diagnostic performance data. To address this need, evidence on the diagnostic performance of RBT as a human brucellosis point-of-care test was reviewed. The review was initially focused on studies conducted in Tanzania but was later extended to worldwide because few relevant studies from Tanzania were identified. Databases including Web of Science, Embase, MEDLINE, and World Health Organization Global Index Medicus were searched for studies assessing the diagnostic performance of RBT (sensitivity and specificity) for detection of human brucellosis, in comparison to the reference standard culture. Sixteen eligible studies were identified and reviewed following screening. The diagnostic sensitivity (DSe) and specificity (DSp) of RBT compared to culture as the gold standard were 87.5% and 100%, respectively, in studies that used suitable “true positive” and “true negative” patient comparison groups and were considered to be of high scientific quality. Diagnostic DSe and DSp of RBT compared to culture in studies that also used suitable “true positive” and “true negative” patient comparison groups but were considered to be of moderate scientific quality varied from 92.5% to 100% and 94.3 to 99.9%, respectively. The good diagnostic performance of RBT combined with its simplicity, quickness, and affordability makes RBT an ideal (or close to) stand-alone point-of-care test for early clinical diagnosis and management of human brucellosis and nonmalarial fevers in small and understaffed health facilities and laboratories in endemic areas in Africa and elsewhere.

## 1. Introduction

Brucellosis is a disease of humans and animals caused by several species of the genus *Brucella* bacteria [1]. *Brucella abortus*, *B. suis*, and *B. melitensis*, three of the so-called smooth *Brucellae*, preferentially infect cattle, swine, and sheep and goats, respectively. These animals are the source of most cases of human brucellosis, a disease that can be debilitating in both acute and chronic phases [2]. Brucellosis infection has economic ramifications due to time lost by patients from normal daily activities [3] and losses in animal production [4]. In endemic African settings, such as Tanzania, human brucellosis often results in an undulating febrile illness, with significant morbidity and mortality in children and adults [5–7].

The incidence of human brucellosis varies widely among and within countries, with a higher disease incidence seen in low- and middle-income countries when compared to high-income countries. In Africa, for example, incidences calculated from two prospective studies in Egypt that involved a surveillance system for acute febrile illness in rural areas ranged from 18 to 70 cases per 100,000 person-years [8, 9]. In Europe and North America, incidence rates are generally much lower. Incidence rates reported for the European Union were 0.08 cases per 100,000 person-years, three quarters of which were reported by Greece, Spain, and Portugal [10], while incidence rates in the USA were 0.02 to 0.09 cases per 100,000 person-years [11]. Variation in incidence is also observed within countries and target population subgroups [12]. In Tanzania, reported human seroprevalence has varied from 0.6 to 15.4% [5, 7, 13, 14], with *B. abortus* and *B. melitensis* most often isolated from affected individuals [7]. The observed variations in reported prevalence in Tanzania [5, 7, 13, 14] are likely related to differences in study areas, study populations, and diagnostic tests, protocols, and reagents used.

Understanding the true burden of human brucellosis in endemic settings, such as Tanzania, has been challenged by delayed diagnosis, underdiagnosis, and underreporting [5–7]. The nonspecific nature of brucellosis symptoms and overlapping clinical manifestations with malaria [6] highlight the importance of identifying reliable point-of-care (diagnostic testing at the time and place of patient care) diagnostic tools to guide clinicians and facilitate detection of human brucellosis in endemic areas. To date, there has been no guidance provided to clinicians and laboratory personnel on the availability and use of point-of-care tests for diagnosis of human brucellosis cases in Tanzania and in many other endemic areas, where both brucellosis and malaria are prevalent. Considering that the disease tends to be more common in rural areas, where clinical and laboratory facilities may be minimally equipped and staffed, selection of suitable point-of-care tests requires evaluation of tests' sensitivity, specificity, ease of use, and affordability.

A definitive diagnosis of *Brucella* spp. infection requires either isolation of the causative organism or the demonstration of high levels of specific antibodies [15]. Bacteriological culture is considered the gold standard but has several drawbacks: it can be time-consuming, requiring 7–10 days for isolation; it has variable sensitivity in acute cases, ranging from 30–90% [16–20], and even lower sensitivity in chronic cases [19, 21]; and the costs and risks to lab staff can be significant [2]. These limitations make culture impractical for endemic areas that are often located in resource-limited settings. Due to the limitations of the culture, laboratory diagnosis of brucellosis, particularly in endemic countries, often relies on detecting specific serum antibodies. Serological tests are easier to execute, and results can be obtained within a short time [2, 22], usually within a few minutes to less than 24 hours depending on the serological test, facilitating diagnosis in rural settings [15]. Serologic assays available for human brucellosis testing include the standard RBT, here after referred to as RBT [13, 14], titrated RBT (involving serum dilutions made in phosphate-buffered saline and then tested with an equal volume of the RBT reagent) [2, 23], competitive ELISA [13], buffered acidified plate antigen test (BAPA), rivanol precipitation test [14], standard microagglutination test (MAT) [5, 6], *Brucella* IgM and IgG ELISA, and serum agglutination test (SAT), also referred to as Wright's test or serum tube agglutination test (STAT) [7].

Compared to most other serological tests, RBT is rapid, easy to use, and relatively affordable as a screening test for *Brucella* spp. infection [2, 15]. The RBT is sensitive for diagnosing *Brucella* spp. infection in certain subpopulations, such as in individuals with acute brucellosis; is able to detect agglutinating and nonagglutinating antibodies [15]; discriminates against agglutinins of low avidity; is not subject to prozone (prozone refers to a phenomenon exhibited by some sera, in which agglutination occurs at higher dilution ranges, but is not visible at lower dilutions) [24]; and compares favourably with the SAT in specificity for detecting antibodies in human sera [25]. The RBT is the recommended rapid screening test for antibodies against the smooth lipopolysaccharide (S-LPS) of the outer cell membrane of *Brucella* spp. but can give false-positive reactions with sera from patients infected with *Yersinia enterocolitica* 0 : 9 or other cross-reactive organisms and from healthy individuals that have had contact with S-LPS of *Brucella* spp. without developing disease [26]. Despite its benefits as a rapid diagnostic test for acute brucellosis in low-resource settings, the RBT can have low sensitivity (high number of false negatives) in cases of chronic disease [27, 28].

Even with its limitations, the above features suggest RBT may be an especially useful test in endemic areas with limited laboratory facilities and access. Further assessment of RBT performance data is needed to facilitate the acceptance and uptake of point-of-care tests in endemic areas, such as Tanzania. Although brucellosis diagnostic testing is currently being conducted at some health facilities in Tanzania using RBT and other tests, there has not yet been formal approval by the government of Tanzania for any point-of-care brucellosis diagnostic test.

Evidence on RBT performance could provide useful information for local health authorities and governments to guide decisions on the appropriate choice of point-of-care tests, facilitate early clinical diagnosis and management of human brucellosis illnesses and other nonmalarial fevers in endemic regions, and contribute to reducing the impact of nonmalarial febrile illnesses.

The purpose of this study was to review available evidence on the diagnostic performance of RBT, with a focus on its potential use as a routine point-of-care test in endemic and resource-limited areas of Africa, with Tanzania as an example. The review was initially focused on studies conducted in Tanzania but was later extended to worldwide because few relevant studies from Tanzania were identified. The specific aims were to review the diagnostic sensitivity (DSe) and specificity (DSp) of RBT compared to culture which is considered the gold standard test.

## 2. Methods

A systematic literature review was conducted, through which published evidence on performance of RBT as a point-of-care test for human brucellosis was collected and assessed. Review of the literature was conducted following the PRISMA guidelines for systematic reviews and meta-analyses [29]. The review took into consideration available published evidence on diagnostic performance of RBT for detection of human brucellosis in studies conducted in Tanzania and worldwide.

### 2.1. Eligibility Criteria

Eligible studies were full-text peer-reviewed manuscripts or scientific expert opinion reports published in English between January 1, 1970, and December 31, 2019, that used either RBT alone or a combination of RBT and other tests to detect *Brucella* spp. infection in humans in Tanzania and globally. Studies that investigated *Brucella* spp. infections in animals only or studies that considered *Brucella* spp. molecular testing or genotyping only were not considered for the purpose of this systematic review.

### 2.2. Search Strategy

First, the PICO (population, intervention, comparator, and outcome) was defined, which guided the definition of the search terms of interest used to identify potentially eligible studies. Population: reports in which *Brucella* spp. were detected in humans in Tanzania and globally as the population of interest was reviewed. Intervention: the study focused on the assessment of diagnostic performance of RBT for detection of *Brucella* spp. in humans, compared to culture as the reference standard. Comparators: the comparators used were other diagnostic tests for human brucellosis (such as culture). Outcomes: the outcome of interest was performance of RBT for detection of *Brucella* spp. in human populations, compared to culture as the reference standard. For the purpose of this review, the following were considered as outcomes: diagnostic sensitivity and specificity.

The search strategy used science database search engines, grey literature websites, and citation tracking to identify potentially relevant studies. Electronic databases including MEDLINE (PubMed), Embase, Web of Science (WOS), and other sources (World Health Organization Global Index Medicus) were searched for relevant studies. Free text searches covered both title and abstract. Searches included Medical Subject Headings (MeSH) thesaurus headings and free text terms that covered the PIO criteria (e.g., population, interventions, and outcomes). The free terms and MeSH headings were combined with Boolean operator OR and/or combined with AND at a later stage of the search process (Table S2). The combinations of search terms across the PIO groups were extracted separately to produce the final list of search hits from each database. Search terms for comparators were not defined as both studies with and without comparators were included in the study.

### 2.3. Study Screening

**Figure 1:**
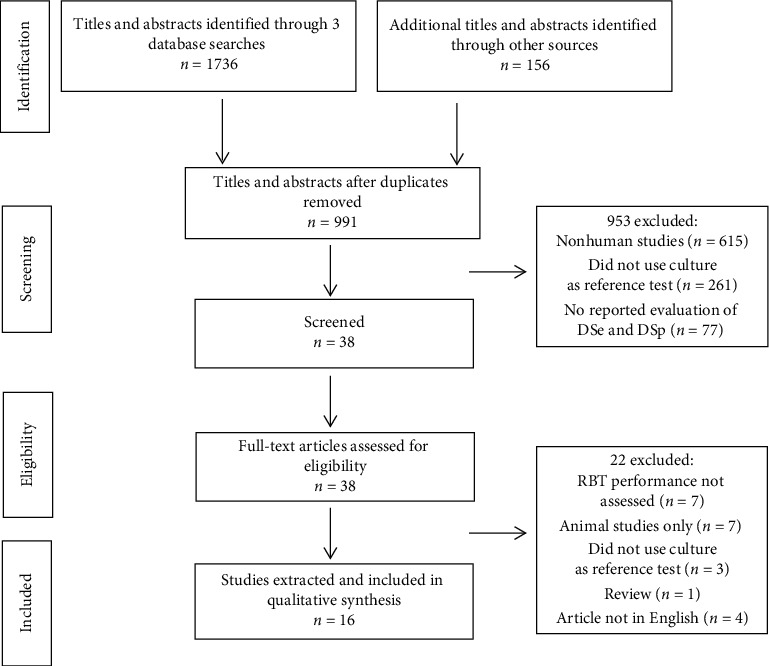
Flow diagram of study selection procedures, adapted from Moher et al. [29]. The number of documents identified and screened was recorded at each stage.

All articles meeting search criteria from the literature were imported into EndNote reference management software, and duplicates were removed prior to the first stage sifting process. All identified studies were then screened for eligibility based on the title, abstract, and full text. The number of documents identified and screened was recorded at each stage and presented in a PRISMA diagram as described by Moher et al. [29] (Figure 1), and reasons for exclusion were noted.

### 2.4. Data Extraction and Synthesis

Following data extraction into an Excel database, study characteristics (e.g., study design, sampling methods, patient groups compared, and diagnostic tests evaluated) and outcomes of interest were described. Data for RBT diagnostic sensitivity and specificity and positive and negative predictive values (where provided) compared to culture were assessed. Data on merits and limitations of RBT were also assessed.

## 3. Results

### 3.1. Search Results

Following the search criteria, literature searches in Web of Science, Embase, and MEDLINE identified 999, 469, and 268 studies, respectively. Additional searches in WHO Global Index Medicus and reference tracking retrieved 149 and 7 studies, respectively, resulting in an overall total of 1892 studies. After removing 901 duplicates, 991 studies were selected for further evaluation based on the title and abstract. Further screening and eligibility assessment resulted in additional exclusions. First, 953 studies were excluded because 615 did not describe use of RBT in humans, 261 studies did not use culture as the reference test, and 77 studies did not evaluate or report diagnostic sensitivity and specificity. The remaining 38 full-text articles were further assessed for eligibility. An additional 22 studies were excluded because RBT diagnostic performance was not assessed (*n* = 7), studies conducted in animals only (*n* = 7), culture was not used as a reference test (*n* = 3), only a narrative review was provided (*n* = 1), or the manuscript was not in English (*n* = 4). Finally, a total of 16 eligible studies were selected for data extraction and synthesis for this review (Figure 1).

### 3.2. Characteristics of the Studies Included in the Review

Key characteristics of the sixteen studies included in the review are summarized in Table 1. Studies were published between 1970 and 2019. Studies were conducted in Spain (*n* = 5), Greece (*n* = 1), Eastern Europe (*n* = 4: Kazakhstan = 1, Bosnia and Herzegovina = 1, Macedonia = 1, and Bulgaria = 1), Turkey (*n* = 3), Egypt (*n* = 1), Kuwait (*n* = 1), and India (*n* = 1).

### 3.3. Reference Test

In 13 of the 16 studies included in this review, standard RBT performance was compared to culture as the reference test [2, 15, 19–21, 23, 27, 28, 30–32], and in 3/16 studies [2, 23, 34], titrated RBT (involving serum dilutions made in phosphate-buffered saline and then tested with an equal volume of the RBT reagent) was compared to culture (Table 1).

### 3.4. RBT Diagnostic Sensitivity and Specificity

In 8 of the 16 studies included in this review, DSe and DSp of RBT compared to culture as the reference test were estimated by the authors and reported [15, 19, 20, 23, 27, 28, 34, 36] (Table 1), and in 8/16 studies, RBT diagnostic sensitivity and specificity were not reported [2, 21, 30–33, 35, 37], but could be estimated independently [2, 30].

### 3.5. Characteristics of Patient Groups Used to Assess RBT Performance

The characteristics of patient groups used to evaluate RBT performance compared to culture varied in the reviewed studies. In some of the reviewed studies, the patient comparison groups were described based on diagnostic criteria of brucellosis and stage/phase of brucellosis, as shown in Table 1. The diagnostic criteria used to define patient comparison groups included the use of historical, clinical, epidemiological, and laboratory (culture, serology) criteria. In some studies, patient groups were also categorised by the stage of brucellosis as acute, subacute, and chronic.

### 3.6. Interpretation of Diagnostic Sensitivity and Specificity of RBT Compared to Culture as the Gold Standard

The use of patient groups that reflect the actual population in which a test is likely to be used is an important consideration for assessment of DSe and DSp. The patient comparison groups can be selected based on relevant diagnostic criteria including historical, epidemiological, clinical, and laboratory data, as well as stage of disease. Another important consideration for assessing DSe and DSp is the use of patient groups considered “true positive” (confirmed brucellosis cases) and “true negative” (confirmed brucellosis-free). For purposes of proper calculation and interpretation of DSe and DSp of RBT (compared to culture as the gold standard),“true positive” patient groups were defined as patients considered to have brucellosis based on culture-positive results and/or clinical and/or epidemiological criteria, and “true negative” patient groups were defined as patients who are considered brucellosis-free based on culture-negative results and/or clinical and/or epidemiological criteria. The reviewed studies were assessed to determine if “true positive” and “true negative” patient groups were used to estimate DSe and DSp of RBT compared to culture as the gold standard. Patient groups that met the definitions of “true positive” or “true negative” were described as either suitable or not suitable, as shown in Table 1.

The reviewed studies were further categorised as high or moderate or poor quality to reflect the scientific quality of the reviewed studies with respect to use of appropriate “true positive” and “true negative” patient groups and proper assessment of DSe and DSp of RBT (compared to culture). High-quality studies were defined as studies in which suitable “true positive” patient groups (based on culture-positive results and clinical and/or epidemiological criteria) and “true negative” patient groups (based on culture-negative results and clinical and/or epidemiological criteria) were used to estimate DSe and DSp of RBT compared to culture. Moderate-quality studies were defined as studies that used a suitable “true positive” patient group (based on culture-positive results and clinical and/or epidemiological criteria) and a suitable “true negative” patient group (based on either culture-negative results or clinical and/or epidemiological criteria). Poor-quality studies were defined as studies that used a suitable “true positive” patient group but did not use a “true negative” patient group or as studies that did not use a suitable “true positive” or “true negative” patient group.

### 3.7. Interpretation of RBT Diagnostic Sensitivity and Specificity in Studies Categorised as High Quality

Of the sixteen studies, only two studies [27, 30] were considered high quality. Although DSe of RBT in the “true positive” group (patients from whom *Brucella melitensis* was isolated) and DSp of RBT in the “true negative” group (patients with fever but no other symptoms of brucellosis) were not directly reported by Saz et al. [30], DSe of the “true positive” group and DSp of the “true negative” group could be estimated independently based on the information provided. DSe of RBT was independently calculated as 87.5% (182/208) using patients from whom *Brucella melitensis* was isolated (*n* = 208). Calculated DSp of RBT was 100% (107/107) using patients with fever but no other symptoms of brucellosis, from whom no *Brucella* spp. were isolated and for whom all conventional tests were negative (*n* = 107).

In [27], DSe of RBT in patients with brucellosis categorised as acute (*n* = 296), subacute (*n* = 44), chronic (*n* = 40), and CNS brucellosis (*n* = 317) was reported as 98%, 84%, 61%, and 22%, respectively [27], and DSp of RBT was 100% in patients used as “controls” (patients with other infectious diseases, noninfectious diseases, and normal healthy individuals, *n* = 345) (Table 2). Reported DSe of RBT should however be interpreted with caution because inadequate information was provided to allow for independent calculation of DSe (compared to culture) in the “true positive” patient group (patients with brucellosis). For example, it is not clear if investigators took into consideration the culture results (as the gold standard) when estimating reported DSe of the RBT, and the number of culture-positive patients that also tested positive on RBT could not be determined to allow for independent calculation of DSe of RBT. Such information would be useful for proper assessment of DSe especially in the chronic brucellosis patients in which DSe was reported to be low (61%).

### 3.8. Interpretation of RBT Diagnostic Sensitivity and Specificity in Studies Categorised as Moderate Quality

Four of the sixteen studies [2, 15, 19, 23] were considered moderate quality. Although DSe of RBT in the “true positive” group (individuals with brucellosis confirmed by culture and defined as short or long evolution) and DSp of RBT in the “true negative” group (patients with no brucellosis symptoms presented for other conditions) were not directly reported by Diaz et al. [2], DSe and DSp could be estimated independently based on the information provided. Independently calculated DSe of RBT in individuals with brucellosis confirmed by culture and defined as short evolution (acute) or long evolution (chronic) was 100% (208/208), and DSp of RBT in patients with no brucellosis symptoms presented for other conditions was 99.9% (1558/1559). Even though the patient group which was used to estimate DSp of RBT (compared to culture) comprised patients with no brucellosis symptoms that presented for diagnosis of other infections and had tested negative for brucellosis using serology (*n* = 1559), this patient group was not tested with culture and cannot be defined as brucellosis-free (if performance of RBT is compared to culture as the gold standard); hence, the categorization of this study is moderate quality (versus high quality).

In [15], DSe of RBT in the “true positive” group (patients with brucellosis) and DSp of RBT in the “true negative” group (patients with different infectious, autoimmune, or neoplastic processes) were not directly reported; DSe in the “true positive” group could not be independently estimated, but DSp for the “true negative” could be estimated based on the information provided. DSe of RBT could not be independently calculated because information was not provided on the number of culture-positive patients (true positive) used in the final analysis. The patient group used to estimate DSe included individuals with brucellosis (*n* = 711), and the reported final analysis included 697 patients, of which 445 tested culture-positive, and 266 were confirmed to have brucellosis based on clinical and serological data. The 697 patients were divided into 3 groups by exposure status, and reported DSe for other patient groups was estimated on this basis (Table 2). The number of culture-positive patients (*n* = 445) that tested RBT-positive was not provided making it difficult to independently calculate DSe. DSp of RBT for the “true negative” group was independently calculated as 94.3% (166/176) using patients with different infectious, autoimmune, or neoplastic processes with a precise aetiological diagnosis but which involved an initial differential diagnosis with brucellosis. Similar to Diaz et al.'s study, even though the patient group used to estimate DSp of RBT (compared to culture) comprised patients with other diagnoses (*n* = 176), this patient group was not tested with the culture and cannot be defined as brucellosis-free (if performance of RBT is being compared to culture as the gold standard); hence, the categorization of this study is moderate quality (versus high quality).

In [23], DSe of titrated RB in the “true positive” group (patients with acute brucellosis) was incorrectly reported as 100% (Table 2). Independent estimation of DSe in this patient group based on culture as the gold standard was 52% (13/25) and not 100% as shown in Table 2 [23]. DSp of titrated RB was not reported for the “true negative” group which included patients classified as healthy individuals (blood donors) (*n* = 90). And even though a patient group with healthy individuals (blood donors, *n* = 90) was used to estimate DSp of titrated RB, this patient group was not tested with culture and cannot be defined as brucellosis-free if performance of RBT is compared to culture as the gold standard; hence, the categorization of this study is moderate quality (versus high quality).

In [19], DSe of RBT in the “true positive” group (patients with primary infection and showing acute clinical symptoms) was incorrectly reported as 100% (Table 2). An independent estimation of DSe of RBT (compared to culture) in the “true positive” group (*n* = 38) revealed a value of 92.5% (35/38), which is slightly lower than the reported 100%. The reported DSp of 99% (Table 2) was based on the “true positive” group (*n* = 38) and not the “true negative” group. DSp of the “true negative” group which included “negative healthy” patients (*n* = 346) could not be independently calculated because information on test results for RBT and culture for this group was not provided by the investigators.

### 3.9. Interpretation of RBT Diagnostic Sensitivity and Specificity in Studies Categorised as Poor Quality

Ten of the sixteen studies [20, 21, 28, 31–37] were considered to be of poor quality; interpretation of DSe of RBT compared to culture in the “true positive” group and DSp in the “true negative” group was difficult or not possible in some studies. In [20], DSe of RBT in the “true positive” group (patients with signs of brucellosis) was incorrectly reported as 100% (91/91), yet the sensitivity of culture was reported as 28/91 (30.8%). These culture results suggest DSe of RBT compared to culture should have been based on the 28 culture-positive patients (28/28 = 100%). Furthermore, there is incomplete and inconsistent information provided on DSe, for example, the sensitivity of culture was reported as 28/91 (30.8%), but another value of 43.8 was also reported [20]. DSp of the “true negative” group could not be independently calculated based on the information provided. The patient group with signs of brucellosis (*n* = 91) was used to estimate both DSe (*n* = 28, culture-positive) and DSp (*n* = 63, culture-negative). Even though 63 patients in this patient group tested negative on the culture, this group was not suitable for estimation of DSp of RBT compared to culture. Proper estimation of DSp requires the use of a suitable patient group (“true negative” comprising patients that are unlikely to have brucellosis and confirmed to be brucellosis-free by testing negative on the culture (or serology, where culture is not feasible). Secondly, all 91 patients with signs of brucellosis were reported as positive on RBT, but it was difficult to confirm if the reported 63 culture-negative patients were also RBT-negative. Considering the poor quality of the reported information and the inability to properly determine DSp of RBT compared to culture, this paper was not reviewed further.

In [28], DSe of RBT in the “true positive” group (patients with clinical suspicion of brucellosis, *n* = 471) was reported but incorrectly. Reported DSe for RBT in patients with clinical suspicion of brucellosis categorised as overall (acute, subacute, and chronic), acute (<6 months of illness) and subacute (6–12 months of illness), and chronic (>1 year of illness) brucellosis was 71.4% (45/63), 94.2 (49/52), and 54.5 (6/11), respectively (Table 2). Culture was performed on a subset of 76 patients, and 82.9% (63/76) were positive on culture. When categorised by stage of brucellosis, the reported positive culture results for the acute group (*n* = 50) were 96.2%. However, if only the 63 patients tested by culture (gold standard) are considered, the sensitivity of culture becomes 50/63 = 79.4% (and not 96.2%). The reported positive culture results for the subacute group (*n* = 2) were 66.7%, but if only the 63 patients tested by culture (gold standard) are considered, then this value becomes 2/63 = 3.2% (and not 66.7%). The reported positive culture results for the chronic group (*n* = 11) were 52.4%, but if only the 63 patients tested by culture (gold standard) are considered, then this value becomes 11/63 = 17.5% (and not 52.4%). In Table 2, an overall DSe of 71.4% is reported for acute, subacute, and chronic patients (*n* = 63), implying an overall total of 45 patients tested culture-positive (45/63 = 71.4%), but how the value of 45 was obtained could not be independently determined. DSp of RBT compared to culture was not assessed, and no “true negative” or negative gold standard population was used to evaluate DSp of RBT. As such, it was not possible to independently determine DSp or fully determine the diagnostic performance of RBT (compared to culture) in this study, and the study was not reviewed further.

In [21], DSe of RBT in the “true positive” group (patients with acute brucellosis) and DSp of the “true negative” group (healthy individuals) were not reported and could not be independently calculated based on the information provided. No culture and RBT test results were reported for patients with acute brucellosis (*n* = 592) that would have been used to estimate DSe and for healthy individuals (voluntary blood donors and people from endemic areas, *n* = 169) that would have been used to estimate DSp. As such, the reported results could not be independently assessed. This study was not reviewed any further because of the poor quality of information and the inability to determine DSe and DSp of RBT compared to culture.

In four studies, Andriopoulos et al. [33], Erdem et al. [35], Ivanov et al. [37], and Yumuk [31], DSe of RBT in the “true positive” groups and DSp of the “true negative” groups were not reported. In addition, “true positive” and “true negative” patient groups were not used, and DSe and DSp could not be independently calculated based on the information provided. In [33], it was not possible to independently estimate DSe and DSp. The patient group used was not a suitable “true positive” patient group because culture was not performed when patients were retested 3–13 years later. Additionally, no “true negative” patient group was used, and no DSp was assessed. In [35], DSe and DSp could not be independently determined based on the information provided. Even though culture was performed in the patient group diagnosed with chronic neurobrucellosis, no information was provided on RBT performance compared to culture. Furthermore, no suitable “true negative” patient group was used, making it impossible to independently calculate DSp. In [37], DSe and DSp could not be independently assessed based on the information provided, and culture was not performed for patients with brucellosis (“true positive” to allow for calculation of DSe) or those considered healthy (“true negative” to allow for calculation of DSp). In [31], the information provided was incomplete to allow for independent calculation of DSe and DSp of RBT compared to culture. Except for the overall group (*n* = 281), where the number of culture-positive patients was reported as 32 (55 RBT-positive patients minus 23 false-positive patients, assuming RBT was compared to culture), it was difficult to determine the number of culture-positive patients in the defined groups. As such, it was not possible to determine if there were suitable “true positive” and “true negative” groups to use for the estimation of DSe and DSp of RBT compared to culture. The above four studies were not reviewed any further because of the poor quality of information and the inability to determine DSe and DSp of RBT compared to culture.

In [32], DSe of RBT in the “true positive” group and DSp of the “true negative” group were not reported. In addition, a “true negative” patient group was not used, and DSe and DSp of RBT compared to culture could not be independently calculated because no or incomplete information was provided. This study was also not reviewed any further because of the poor quality of information and the inability to determine DSe and DSp of RBT compared to culture.

In [34], DSe of RBT compared to culture as the gold standard was incorrectly calculated and reported as 88.9% (Table 2). An independent calculation showed DSe to be 91.1% (51/56) based on information provided by the authors [34]. DSp of the “true negative” group was not reported, and a “true negative” patient group was not used. The authors reported DSp of RBT of 87.7% but did provide information to indicate if a suitable “true negative” patient group was used to estimate DSp. Based on information provided, it appears patients with clinical signs of brucellosis (*n* = 200) were used to estimate DSp of RBT compared to culture. This approach would not be appropriate for estimation of DSp (using culture as the gold standard) because such patients would not be considered brucellosis-free given the presence of clinical signs of brucellosis even without testing. This study was not reviewed any further because of the poor quality of information and the inability to properly estimate DSp of RBT compared to culture.

In [36], DSe of RBT in the “true positive” group was reported but incorrectly. A suitable “true positive” patient group which comprised patients with clinical suspicion of brucellosis (*n* = 50) was used to estimate RBT performance compared to culture (gold standard) but had very few culture-positive patients (*n* = 6), which does not allow for proper assessment of DSe. Reported DSe for RBT (20/20, 100%, Table 2) was incorrectly calculated by authors; the calculation was based on the 6 culture-positive and 14 SAT-positive patients (instead of only the 6 culture-positive patients). DSp of the “true negative” group was not reported, and a suitable “true negative” patient group was not used to calculate DSp of RBT performance (compared to culture as the gold standard). DSp of RBT was estimated using patients with clinical suspicion of brucellosis (*n* = 30), and it could not be independently determined if this group of patients tested culture-negative. This study was not reviewed any further because of the poor quality of information and the inability to properly estimate DSe and DSp of RBT compared to culture.

## 4. Discussion

The performance of RBT for diagnosing human brucellosis was reviewed in sixteen studies, and findings indicated that RBT can reliably detect *Brucella* spp. exposure in patient subpopulations likely to be found in endemic areas and at different stages of brucellosis illness. The scientific quality of the reviewed studies varied widely with respect to suitability of patient groups used to estimate DSe and DSp of RBT and the level of detail and quality of data provided, which subsequently impacted the interpretation of reported DSe and DSp of RBT (compared to culture as the gold standard) and the ability to independently estimate DSe and DSp. Only six of the sixteen reviewed studies were considered to be high or moderate scientific quality and were discussed further. Ten of the sixteen studies were considered to be of poor scientific quality; the information provided did not allow for proper estimation and interpretation of DSe and DSp of RBT (compared to culture as the gold standard), and these studies were not discussed further.

### 4.1. Interpretation of RBT Diagnostic Sensitivity and Specificity in Studies Categorised as High Quality

In the two studies considered to be of high scientific quality, DSe and DSp of RBT compared to culture as the gold standard were 87.5% and 100%, respectively [27, 30]. In [30], suitable “true positive” (based on culture-positive results and clinical and/or epidemiological criteria) and “true negative” (based on culture-negative results and clinical and/or epidemiological criteria) patient groups were used which allows for proper estimation of DSe and DSp of RBT compared to culture. DSe and DSp of RBT were not reported but could be estimated independently based on the information provided. Following independent assessment, DSe of RBT compared to culture was 87.5% (182/208) in patients from whom *Brucella melitensis* was isolated (*n* = 208), and DSp was 100% (107/107) in patients with fever but no other symptoms of brucellosis from whom no *Brucella* spp. were isolated and for whom all conventional tests were negative (*n* = 107).

In [27], suitable “true positive” (based on culture-positive results and clinical and/or epidemiological criteria) and “true negative” (based on culture-negative results and clinical and/or epidemiological criteria) patient groups were also used which allows for proper estimation of DSe and DSp of RBT compared to culture. Reported DSe of RBT in patients with brucellosis categorised as acute, subacute, chronic, and neurobrucellosis was 98%, 84%, 61%, and 22%, respectively, and DSp was 100% in patients with other infectious diseases, noninfectious diseases, and normal healthy individuals. However, incomplete information was provided on the number of culture-positive patients that tested positive on RBT which made it difficult to independently calculate DSe of RBT (compared to culture) in different patient groups. Reported DSe of RBT in this study should therefore be interpreted with caution.

### 4.2. Interpretation of RBT Diagnostic Sensitivity and Specificity in Studies Categorised as Moderate Quality

In the four studies considered to be of moderate scientific quality, DSe and DSp of RBT compared to culture varied from 92.5% to 100% and 94.3 to 99.9%, respectively [2, 15, 19, 23]. In all four studies, suitable “true positive” (based on culture-positive results and clinical and/or epidemiological criteria) and “true negative” (based on either culture-negative results or clinical and/or epidemiological criteria) patient groups were used to estimate DSe and DSp of RBT compared to culture.

In [2], DSe and DSp of RBT compared to culture were not directly reported, but DSe was independently calculated as 100% (208/208) in individuals with brucellosis confirmed by culture and defined as short evolution (acute) or long evolution (chronic), and DSp of RBT in patients with no brucellosis symptoms presented for other conditions was 99.9% (1558/1559).

In [15], DSe of RBT in the “true positive” patient group (individuals with brucellosis confirmed with culture) could not be independently assessed because information was not provided on the number of culture-positive patients (*n* = 445) that tested RBT-positive. DSp of RBT for the “true negative” group was correctly reported and also independently calculated as 94.3% (166/176) using patients with different infectious, autoimmune, or neoplastic processes.

In [23], DSe of titrated RB in the “true positive” group (patients with acute brucellosis) was incorrectly reported as 100%, and independent estimation of DSe in the same patient group compared to culture as the gold standard was lower at 52% (13/25). The low number of patients with acute brucellosis (*n* = 25) used to estimate DSe was of concern because it could have impacted the DSe value. Therefore, DSe of RBT in this study should be interpreted with caution. Although a “true negative” group, which included patients classified as healthy individuals (blood donors) (*n* = 90), was used, DSp of titrated RB was not reported.

In [19], DSe of RBT was incorrectly reported as 100% in the “true positive” group which included patients with acute brucellosis (*n* = 38). An independent estimation of DSe of RBT (compared to culture) in the same “true positive” group revealed DSe of 92.5% (35/38), which is slightly lower than the reported 100%. DSp of RBT compared to culture using a “true negative” patient group could not be independently determined. Although a “true negative” group, which included “negative healthy” patients (*n* = 346), was used, DSp of RBT could not be independently calculated because information on test results for RBT and culture for the “negative healthy” group (*n* = 346) was not provided.

It is important to note that even though the patient groups used to estimate DSp of RBT (compared to culture) in the studies by Diaz et al. [2], Ruiz-Mesa et al. [15], Gomez et al. [23], and Serra and Vinas [19] did not have brucellosis symptoms [2, 15, 19, 23] and tested negative for brucellosis on serology [15], these patient groups were not tested using culture [2, 15, 19, 23] and could not be defined as brucellosis-free if DSp of RBT was compared to culture as the gold standard.

Worthwhile noting as well that although the studies by Diaz et al. [2], Ruiz-Mesa et al. [15], Gomez et al. [23], and Serra and Vinas [19] were all categorised as moderate scientific quality, the study by Diaz et al. [2] was of better quality compared with the other three studies. Unlike the other three studies [15, 19, 23] where only DSe or DSp could be independently assessed, in Diaz et al.'s study [2], both DSe and DSp of RBT were independently assessed based on information provided.

### 4.3. Considerations of RBT Use in Endemic Settings

Findings from the reviewed studies that properly assessed DSe and DSp of RBT compared to culture (as the gold standard) suggest RBT is an excellent screening test that could provide needed clinical diagnostic support in endemic areas where febrile patients are currently only screened for malaria. Additionally, RBT has other merits, which are particularly relevant in endemic settings: it is inexpensive, simple, and easy to use, requires minimal infrastructure or local preparation, and can be used in small, clinic-based laboratories. The above combined diagnostic and logistic merits suggest RBT should be considered as a rapid point-of-care test for human brucellosis in endemic areas.

### 4.4. Study Limitations

A number of limitations were identified while performing this review. First, few studies exist that evaluate RBT performance compared to the recognized reference standard, culture. Initially, this study was designed to assess studies investigating RBT performance in Tanzania, but the scope was extended to global level following scope searches that identified few relevant studies from this region. Second, in some studies, detailed information on patient comparison groups, test results for culture and RBT, and on DSe and DSp of RBT compared to culture was either incomplete or not reported or not specified by the patient group, which made it difficult to independently assess DSe and DSp of RBT compared to culture. Third, ten of the sixteen studies included in this review did not use suitable “true positive” and/or “true negative” patient groups to assess DSe and DSp of RBT compared to culture. Failure to use appropriate comparison groups while evaluating RBT performance can weaken the internal validity of a study and the inferences drawn. Comparison groups selected based on diagnostic criteria (historical, clinical, epidemiological, and laboratory data) and stage of disease are useful in reflecting the actual population in which the test is likely to be used. And finally, it is difficult to make proper comparisons of RBT performance across studies based on the stage of illness (acute, subacute, and chronic) because the definitions used varied and were subjective. Categorizing of patient groups by stage of illness is important because clinical course can influence the detection of brucellosis.

A knowledge gap identified as part of this review was that high-quality data on the true burden of human brucellosis and estimates of brucellosis disability-adjusted life years (DALY) calculations are limited, even more so for subpopulations in endemic settings, such as Tanzania. Brucellosis DALY data are useful for assessing disease burden and informing policy geared to human brucellosis control and prevention. Estimates of disability weights have been proposed for DALY calculation, using Mongolian patient data [4] and systematic review and meta-analysis data [38]. Further studies that examine the accuracy of these estimates in subpopulations with varying exposures in endemic settings are necessary to confirm suitability for assessment of brucellosis disease burden.

## 5. Conclusion

In conclusion, the good diagnostic performance combined with its simplicity, quickness, and affordability makes RBT an ideal (or close to) stand-alone point-of-care test for early clinical diagnosis and management of human brucellosis and nonmalarial fevers in small and understaffed health facilities and laboratories in endemic areas in Africa and elsewhere.

## Figures and Tables

**Table 1:** Summary of published studies that investigated the performance of Rose Bengal test compared to culture as the reference test for diagnosis of human *Brucella* spp. infection.

Study	Diagnostic tests investigated	RBT DSe and DSp reported	Patient groups used to assess RBT performance	Diagnostic criteria used to select patient groups	“True positive” or “true negative” patient group used^*∗∗*^	Country
Diaz et al. [2]	RBT, titrated RBT^*∗*^, SAT, Brucellacapt, Coombs, LFiC-IgM and IgG, culture.	No, but DSe and DSp can be calculated from values provided.	(1) Individuals with brucellosis confirmed by culture (*n* = 208), included a subset of patients broadly categorised as acute and chronic based on IgM and IgG profile determined by LFiC.	Clinical findings, laboratory criteria (culture, serology).	Suitable “true positive” patient group used. Based on culture-positive results and clinical and/or epidemiological criteria.	Spain
			(2) Individuals with professional contact with infected animals or products or accidentally injected with *Brucella* vaccine, but asymptomatic (*n* = 20).	Clinical findings, laboratory and epidemiological criteria.	NA	
			(3) Brucellosis patients showing prozone effect (*n* = 11).			
			(4) Patients with no brucellosis symptoms (serum sent to the lab for diagnosis of other infections) (*n* = 1559).	Clinical findings.	Suitable “true negative” patient group used. Even though patients had no brucellosis symptoms, no culture results were reported. Diagnosis based only on clinical and/or epidemiological criteria.	

Gomez et al. [23]	Titrated RBT^*∗*^, MAT, Brucellacapt, ELISA IgG, IgM and IgA, culture	Yes, but patient group used to calculate DSp was unsuitable.	(1) Patients with acute brucellosis (*n* = 25).	Clinical findings and either positive culture or serology results (SAT ≥ 160).	Suitable “true positive” patient group used. Based on positive culture results and clinical findings.	Spain
			(2) Healthy individuals (blood donors) (*n* = 90).	Clinical findings (blood donors) and serology results.	Suitable “true negative” patient group used. Even though patients were considered “healthy,” culture was not performed. Diagnosis was based only on clinical findings and serology.	

Mizanbayeva et al. [28]	RBT, SAT, LFA IgM and IgG, culture.	Yes, DSe reported, but DSp was not assessed.	Patients with clinical suspicion of brucellosis (*n* = 471). Patients divided into 3 categories: acute disease (<6 months of illness, *n* = 396), subacute (6–12 months of illness, *n* = 30), and chronic (>1 year of illness, *n* = 43). Culture performed on a subset of 76 patients, and 63/76 were culture-positive.	Clinical findings, laboratory findings (culture, serology).	Suitable “true positive” patient group used. Based on culture-positive results, clinical and/or epidemiological criteria.	Kazakhstan
					No “true negative” patient group assessed.	

Ruiz-Mesa et al. [15]	RBT, culture	Yes, DSp can be calculated from values provided, but DSe for culture-positive patients could not be calculated.	(1) Individuals with brucellosis confirmed with culture (*n* = 711). A subset of patients with positive culture results (*n* = 445).	Laboratory (culture or serology) and clinical criteria.	Suitable “true positive” patient group used. Based on positive culture results and clinical findings.	Spain
			(2) Patients with different infectious, autoimmune, or neoplastic processes with a precise aetiological diagnosis, but which involved an initial differential diagnosis with brucellosis (*n* = 176). This was considered by authors as one of three control groups	Clinical criteria, serology done but not specified if done for this group.	Suitable “true negative” patient group used. Even though patients were not diagnosed with brucellosis, culture was not performed.	
			(3) Individuals exposed repeatedly to *Brucella* spp. during work (*n* = 68). Considered one of three control groups.	Clinical and epidemiological criteria, serology done but not specified if done for this group.	NA. Culture was not performed, and patient group was exposed to brucellosis.	
			(4) Asymptomatic patients with history of brucellosis who had received appropriate treatment and shown no evidence of relapse after 1 year (*n* = 26). Considered one of three control groups.	History, clinical, and epidemiological criteria, serology done but not specified if done for this group.	NA. Culture was not performed, and patient group had a history of brucellosis.	

Saz et al. [30]	RBT, ELISA, SAT, Coombs, culture.	No, but DSe and DSp can be calculated from values provided.	(1) Patients from whom *Brucella melitensis* was isolated (*n* = 208).	Laboratory criteria (culture and serology).	Suitable “true positive” patient group used. Based on positive culture results and clinical findings.	Spain
			(2) Patients with suspected brucellosis and positive results by ≥two conventional tests (*n* = 177).	Clinical findings and laboratory criteria (serology—positive RBT, SAT ≥ 80 and ≥160, Coombs).	NA. Culture was not performed.	
			(3) Patients with fever but no other symptoms of brucellosis, from whom no *Brucella* spp. were isolated and for whom all conventional tests were negative (*n* = 107).	Based on clinical findings, negative culture and serology results (ELISA).	Suitable “true negative” group used. Based on negative culture results, serology and clinical findings.	

Serra and Vinas [19]	RBT, SAT, Coombs, ELISA IgG and IgM, CFT, culture.	Yes, DSe of the “true positive” group could be calculated independently, but DSp of the “true negative” group could not be calculated based on the information provided.	(1) Patients with primary infection (no personal history of brucellosis) and showing acute clinical symptoms (*n* = 38).	Based on either (1) positive culture and serology results (SAT ≥ 160) and clinical evidence, or (2) clinical evidence and positive serology (Coombs test).	Suitable “true positive” group used. Based on positive culture results and clinical criteria.	Spain
			(2) Individuals living in the same area examined (*n* = 346). This group was considered a “negative-healthy” population and used as a control group and compared with the above group (patients with primary infection).	No information provided on laboratory or epidemiological criteria.	Suitable “true negative” patient group used. Even though patients were considered “healthy,” culture results were not reported. Based on clinical findings only.	
			(3) Patients with evidence of previous infection (*n* = 24) based on (i) brucellosis being diagnosed previously or (ii) epidemiological data compatible with long exposure (such as in farmers and veterinarians) and an immune response of “secondary type” (IgG predominating).	Based on positive culture and serology results, clinical and epidemiological criteria.	NA. Patients with a history of brucellosis infection, and culture was performed.	
			(4) Healthy individuals in whom brucellosis had previously been diagnosed and subsequently treated more than 2 years before, with no subsequent symptoms of the disease (“cured” population) (*n* = 55). This group was used as a control group and compared with the above group (patients with evidence of previous infection).	Based on clinical and epidemiological criteria.	Not suitable “true negative” group. Culture was not performed, and individuals had a history of brucellosis.	

Sisirak and Hukić [20]	RBT, ELISA IgM and IgG, culture.	Yes, but DSp could not be calculated independently.	Patients with signs of brucellosis at presentation (*n* = 91). Though not clearly presented, patients were divided into stages of illness based on the duration of illness.	Laboratory criteria (culture and/or serology), clinical findings.	Suitable “true positive” group. Based on positive culture results and clinical criteria.	Bosnia and Herzegovina
					Not suitable “true negative” group used. Unclear information provided on culture and RBT results.	

Taleski [21]	RBT, Coombs, 2-ME, CFT, Indirect ELISA, cELISA, FPA, PCR, culture.	No, DSe and DSp were not reported and could not be calculated independently.	(1) Patients with acute brucellosis (*n* = 592). Diagnosis based on epidemiological data, clinical findings, and laboratory tests.	Epidemiological data, clinical findings, and laboratory tests.	Not suitable “true positive” group. No information provided on culture and RBT results for the group.	Macedonia
			(2) Healthy, voluntary blood donors (*n* = 100). Diagnosis based on epidemiological data, clinical findings, and laboratory tests.	Epidemiological data, clinical findings, and laboratory tests.	Not suitable “true negative” group. No information provided on culture and RBT results for the group.	
			(3) Healthy people from endemic areas (*n* = 69). Diagnosis based on epidemiological data, clinical findings, and laboratory tests.	Epidemiological data, clinical findings, and laboratory tests.	Not suitable “true negative” group. No information provided on culture and RBT results for the group.	

Yumuk et al. [31]	RBT, culture.	No, information provided is incomplete to allow for independent calculation of DSe and DSp.	Patients with clinical signs of brucellosis at presentation. Enrolled if *Brucella* spp. positive on culture. Patients divided into two groups: (1) antinuclear antibody- (ANA-) positive group (*n* = 211); 209 patients with autoimmune, infectious, or neoplastic condition and differential diagnosis of brucellosis and 2 patients with brucellosis and (2) ANA-negative group (*n* = 70); 30 patients with brucellosis but with differential diagnosis of autoimmune disease and 40 healthy individuals.	Clinical findings and laboratory criteria (culture).	Not suitable “true positive” group. Information provided on culture, and RBT results are unclear.	Turkey
					Not suitable “true negative” group. Information provided on culture, and RBT results are not clear.	

Mert et al. [32]	RBT, STAT, culture	No, information provided is incomplete to allow for independent calculation of DSe and DSp.	(1) Patients with culture-positive brucellosis (*n* = 30).	Clinical findings, laboratory criteria (culture, serology).	Suitable “true positive” patient group. Culture performed, but incomplete information provided on culture and RBT results.	Turkey
			(2) Patients with diseases that mimic brucellosis clinically (military tuberculosis, malaria, typhoid fever, adult-onset Still's disease, systemic lupus erythematosus, rheumatoid arthritis, sarcoidosis, and active lymphoma) (*n* = 280).	Clinical findings, laboratory criteria (serology).	Not suitable “true negative group” used. No information provided on culture and RBT results for this group.	

Andriopoulos et al. [33]	RBT, STA, culture	No, DSe and DSp were not assessed because no suitable patient groups were used.	(1) Patients with a history of diagnosis and treatment for brucellosis (*n* = 83), and 72/83 of these patients were considered chronic/relapsing cases and located and retested 3–13 years after first infection and used for analysis. All cases had acute brucellosis on first infection, but chronic brucellosis cases did not have signs of acute brucellosis illness on presentation the second time.	Based on clinical findings and serology	Not suitable “true positive” patient group because culture was not performed when patients were retested. And DSe was not assessed.	Greece
					No “true negative” patient group used and no DSp assessed.	

Mantur et al. [34]	Titrated RBT, SAT, 2-ME, culture.	Yes. DSe and DSp were not assessed correctly.	Patients presented with clinical signs of brucellosis (*n* = 200). Patients were divided into three groups on the basis of the duration of illness—acute (<8 weeks, *n* = 179), subacute (>8 weeks but <52 weeks, *n* = 9), and chronic (>52 weeks, *n* = 12).	Clinical features, epidemiological evidence, and serology and culture considered for presumptive clinical diagnosis of brucellosis.	Suitable “true positive” patient group used; culture performed in a subset of 56 patients.	India
					No “true negative” patient group used and no DSp assessed	

Erdem et al. [35]	RBT, STA, culture.	No. DSe and DSp were not assessed. No suitable patient groups were used.	Patients with a diagnosis of chronic neurobrucellosis (brucellar meningitis or meningoencephalitis).	Clinical features and laboratory findings (serology and culture).	Not suitable “true positive” patient group defined and used.	Turkey
					No “true negative patient group used and no DSp assessed	

Marei et al. [36]	RBT, SAT, 2-ME, LFA IgM and IgG, PCR, culture.	Yes, but DSe and DSp incorrectly calculated and DSp based on unsuitable patient group.	Patients presented with clinical suspicion of brucellosis (*n* = 50).	Brucellosis diagnosis based on clinical findings confirmed by either a positive blood culture or presence of specific serum antibodies (SAT titer ≥ 1/160).	Suitable “true positive” patient group used but culture-positive patients were few (*n* = 6).	Egypt
					Not suitable “true negative” patient group used. Patients had signs of brucellosis and were not clear if culture was negative.	

Ivanov et al. [37]	RBT, SAT, Coombs, ELISA IgM and IgG, culture.	No	Patients with and without clinical signs of brucellosis (*n* = 63). Patients with brucellosis (*n* = 21) categorised as acute (duration up to 12 months, *n* = 6) and chronic (more than 12 months, *n* = 15), and others were healthy (*n* = 42).	Clinical picture and serology.	Not suitable “true positive” patient group used. Culture not performed.	Bulgaria
					No “true negative” patient group used. Culture not performed on “healthy” group and no DSp assessed.	

Araj et al. [27]	ELISA (IgG, IgM, IgA), RBT, culture, MAT, SA.	Yes	Patients with brucellosis (*n* = 380), categorised into acute brucellosis (duration < 2 months, *n* = 296), subacute brucellosis (duration of 2 months to 1 year, *n* = 44), and chronic (duration > 1 year, *n* = 40). Other patient groups used included patients with central nervous system brucellosis: patients with CNS brucellosis (*n* = 45) and patients without CNS brucellosis (*n* = 66); patients with meningitis not caused by *Brucella* (*n* = 62) and patients without meningitis (*n* = 144).	Clinical picture, laboratory findings (serology, culture).	Suitable “true positive” patient group used. Based on culture-positive results and clinical findings.	Kuwait
			Patients used as “controls” (*n* = 345) included: patients with other infectious diseases (*n* = 118), patients with noninfectious diseases (*n* = 20), and normal healthy individuals (*n* = 207).	Clinical picture, laboratory findings (serology, culture).	Suitable “true negative” patient group used. Based on culture and clinical findings.	

DSe: diagnostic sensitivity, DSp: diagnostic specificity, SAT: serum agglutination test, STAT: standard tube agglutination test, STA: Wright standard tube agglutination, Coombs: antihuman globulin test, FPA: fluorescent polarization assay, LFA: lateral flow assay, LFiC: lateral flow immunochromatography assay, MAT: microagglutination test, Brucellacapt: immunocapture-agglutination test, 2-ME: 2-mercaptoethanol test, CFT: compliment fixation test, SA: *Brucella melitensis*-stained antigens. ^*∗*^Titrated RBT: serum dilutions made in phosphate-buffered saline and then tested with an equal volume of RBT reagent. ^*∗∗*^“True positive” and “true negative groups: “true positive” patient group is defined as patients considered to have brucellosis based on culture-positive results and/or clinical and/or epidemiological criteria. “True negative” patient group is defined as patients considered to be brucellosis-free based on either culture-negative result or clinical and/or epidemiological data. NA: additional patient comparison group used to assess DSe and DSp but not considering “true positive” or “true negative patient groups.

**Table 2:** Reported sensitivity and specificity of RBT in patient groups used in reviewed studies.

Patient groups	Test	RBT positive, *n*/*N*	DSe	DSp
*Patients with acute brucellosis (diagnosis of brucellosis was based on clinical findings and on either positive blood cultures for Brucella spp. or the presence of serum antibodies (SAT titer => 160))* [23].	Titrated RBT	NR	100	97
	MAT	NR	92	100
	Brucellacapt	NR	100	100
	IgG ELISA	NR	84	100
	IgM ELISA	NR	60	100
	IgA ELISA	NR	96	98

*Patients with clinical suspicion of brucellosis* [28].	RBT (acute + subacute)	49/52	94.2	NS
	RBT (chronic)	6/11	54.5	NS
	RBT (total)	45/63	71.4	NS
	SAT ≥ 1 : 200 (acute + subacute)	26/52	50	NS
	SAT ≥ 1 : 200 (chronic)	6/11	54.5	NS
	SAT ≥ 1 : 200 (total)	32/63	50.8	NS
	LFA IgM and/or IgG (acute + subacute)	52/52	100	NS
	LFA IgM and/or IgG (chronic)	11/11	100	NS
	LFA IgM and/or IgG (total)	63/63	100	NS
	LFA IgM (acute + subacute)	47/52	90.4	NS
	LFA IgM (chronic)	8/11	72.7	NS
	LFA IgM (total)	55/63	87.3	NS
	LFA IgG (acute + subacute)	39/52	75	NS
	LFA IgG (chronic)	11/11	100	NS
	LFA IgG (total)	50/63	79.4	NS

*Patients presented with clinical signs of brucellosis; clinical features along with epidemiological evidence were considered for presumptive clinical diagnosis of brucellosis* [34].	Titrated RBT	78/200	88.9	87.7

*Patients presented with clinical suspicion of brucellosis* [36].	RBT	20/20	100	100
*Patients with diseases mimicking brucellosis clinically* [36].	RB screening test	0/280	NS	NS
	STAT (≥1/160)^b^	0/280	NS	NS

*Patients with signs of brucellosis at presentation* [20].	RBT	91/91	100	NS
	ELISA IgM	59/91	64.8	NS
	ELISA IgG		56.1	NS

*Patients with signs of brucellosis at presentation* [31].	RBT	NS	NS	NS

*Healthy, voluntary blood donors* [21].	RBT	NS	NS	NS
	Coombs	NS	NS	NS
	2-ME	NS	NS	NS
	CFT	NS	NS	NS
	Indirect ELISA	NS	NS	NS
	cELISA	0/10	NS	NS
	FPA	NS	NS	NS
	PCR	0/30	NS	NS
*Healthy people from endemic areas* [21].	RBT	NS	7	NS

*Individuals with no history of brucellosis or regular exposure to Brucella spp. (patients with different infectious, autoimmune, or neoplastic processes with a precise aetiological diagnosis, but which involved an initial differential diagnosis with brucellosis)* [15].	RB screening test	288/307	93.8	94.3
*Patients with a previous history of brucellosis/asymptomatic individuals infected with Brucella who had received appropriate treatment during the previous 12 months* [15].	RB screening test	49/51	96.1	76.9
*Asymptomatic individuals exposed repeatedly to Brucella infection during their work* [15].	RB screening test	311/339	91.7	94.3

*Patients with primary infection (no personal history of brucellosis) and showing acute clinical symptoms* [19].	RB screening test^c^	38/38	100	99
	Tube agglutination test (≥1/160)	NS	97	99
	Coombs (≥1/320)	NS	100	98
	ELISA IgG	NS	78	83
	ELISA IgM	NS	94	98
	CFT	NS	91	99

*Patients with evidence of previous Brucella infection, with either brucellosis diagnosed previously or epidemiological data compatible with long exposure and an immune response of “secondary type” (IgG predominating on IgM)* [19].	RB screening test	22/24	92	52
	Tube agglutination test (≥1/160)	NS	20	98
	Coombs (≥1/320)	NS	100	80
	ELISA IgG	NS	100	70
	ELISA IgM	NS	54	76
	CFT	NS	88	78

*Patients with acute brucellosis (presented with fever and had nonspecific symptoms such as headache, malaise, arthralgia, and low back pain. Other symptoms included splenomegaly, lymphadenitis, and localization of the disease including genitourinary hematological and osteoarticular involvement)* [33].	RBT	83/83	100	87.5
*Patients with chronic/relapsing brucellosis (defined as patients located 3–13 years following first hospital visit when they presented with acute brucellosis infection). Chronic brucellosis cases did not have signs of acute brucellosis illness on presentation the second time* [33].	RBT	9/72	NS	NS

*Patients categorised as acute (duration up to 12 months)/chronic (more than 12 months)/healthy* [37].	RBT	3/21	NS	NS
*Patients categorised as acute (duration up to 12 months)/chronic (more than 12 months)/healthy* [37].	RBT	3/21	NS	NS

*Patients with a diagnosis of chronic brucellar meningitis or meningoencephalitis* [35].	RBT	118/123	NS	NS
	RBT	75/106	NS	NS
*Patients with fever but no other symptoms of brucellosis and tested negative on culture and other tests* [35].	RB screening test	0/107	NS	NS
	SAT (≥1/180)	0/107	NS	NS
	Coombs (≥1/160)	0/107	NS	NS
	ELISA IgG	NS	NS	NS
	ELISA IgM	NS	NS	NS
	ELISA IgA	NS	NS	NS
	ELISA T^d^	4/107	NS	NS

*Contacts with no brucellosis and had professional contact* [2].	RB screening test^e^	19/20	NS	NS
	RB titration test (>1 : 4)^e^	0/20	NS	NS
	SAT (=160)^e^	3/20	NS	NS
	Brucellacapt (≥320)^e^	8/20	NS	NS
	Coombs^e^	16/20	NS	NS
	LFiC-IgM^e^	4/20	NS	NS
	LFiC-IgG^e^	8/20	NS	NS
*Patient with no recent contact and no symptoms of brucellosis sent to the lab for diagnosis of other infections* [2].	RB screening test^f^	1/1559	NS	NS

*Patients with brucellosis categorised as acute (n* *=* *296)* [27].	RBT		98	100
*Patients with brucellosis categorised as subacute (n* *=* *44)* [27].	RBT		84	100
*Patients with brucellosis categorised as chronic (n* *=* *40)* [27].	RBT		61	100
*Patients with brucellosis categorised as CNS brucellosis (n* *=* *317)* [27].	RBT		22	100
*Patients with other infectious diseases, noninfectious diseases, and normal healthy individuals (n* *=* *345)* [27].	RBT			100

RBT positive: Rose Bengal test positive, DSe: diagnostic sensitivity, DSp: diagnostic specificity, PPV: positive predictive value, NPV: negative predictive value, and NS: not specified. RBT: standard RBT, titrated RBT: titrations made by serial dilutions of serum with saline solution, SAT: serum agglutination test, LFA: lateral flow assay, MAT: microagglutination test, Brucellacapt: immunocapture-agglutination test, LFiC: lateral flow immunochromatography assay. ^b^STAT: only low titres were obtained in 3 patients, one with malaria (1/40), one with lymphoma (1/40), and another with typhoid fever (1/20). ^c^Reported test values were combined values for patients with primary infection (no personal history of brucellosis) showing acute clinical symptoms and patients with evidence of previous infection by *Brucella.*^d^ELISA *T*: detects total specific antibodies. ^e^Sensitivity and specificity not reported, but number of positives in this patient group reported for each test performed. This group comprised 20 people that had had professional contact (veterinarians, slaughterhouse workers, shepherds, etc.) with *B. melitensis*-infected animals or their products or had accidentally injected themselves with vaccine *B. melitensis* Rev 1 and that were followed for a period of at least two years [2]. ^f^Sensitivity and specificity not reported, but number of positives in this patient subgroup reported for standard RBT (RB screening test).

## Supplementary Materials

Supplementary Materials Supplementary 1: S1 Table: list of articles screened at the last stage but excluded and reasons for exclusion. Supplementary 2: S2 Table: search terms used for titles and abstracts and free full text for MEDLINE, Embase, and Web of Science.
