# Supplementary material for: Utility of the Rose Bengal Test as a Point-of-Care Test for Human Brucellosis in Endemic African Settings: A Systematic Review
Source: J Trop Med. 2020 Sep 16;2020:6586182. doi: 10.1155/2020/6586182 (PMC7519193; doi:10.1155/2020/6586182)
Supplement: Supplementary Materials — Supplementary 1: S1 Table: list of articles screened at the last stage but excluded and reasons for exclusion. Supplementary 2: S2 Table: search terms used for titles and abstracts and free full text for MEDLINE, Embase, and Web of Science. [file 6586182.f1.pdf]

Supporting Information

S1 Table. List of articles screened at last stage but excluded and reasons for exclusion.

S2 Table. Search terms used for titles and abstracts and free full text for MEDLINE, EMBASE, WEB OF SCIENCE.

Supporting Information

S1 Table. List of articles screened at last stage but excluded and reasons for exclusion

Author	Study title	Reasons for exclusion
Tiwari *et al* 2013	Development and Comparative Evaluation of a Plate Enzyme-Linked Immunosorbent Assay Based on Recombinant Outer Membrane Antigens Omp28 and Omp31 for Diagnosis of Human Brucellosis.	RBT performance was not assessed in comparison with culture.
Thavaselvam *et al* 2010.	Cloning and expression of the immunoreactive Brucella melitensis 28 kDa outer-membrane protein (Omp28) encoding gene and evaluation of the potential of Omp28 for clinical diagnosis of brucellosis.	RBT performance was not assessed in comparison with culture.
Morales-García *et al* 2014	Clinical, serological and polymerase chain reaction follow-up of a family with brucellosis.	Article not in English and study team was not able to translate.
Lepe *et al* 2001	Detection of Brucella melitensis by BACTEC 9050 system.	Article not in English and study team was not able to translate.
Moreno *et al* 1992	Diagnosis of brucellosis in an endemic area - evaluation of routine diagnostic-tests.	Article not in English and study team was not able to translate.
Gandara *et al* 1994	An evaluation of the effectiveness of laboratory diagnostic methods for brucellosis.	Article not in English and study team was not able to translate.
Gusi *et al* 2019	Comparative performance of lateral flow immunochromatography, iELISA and rose Bengal tests for the diagnosis of cattle, sheep, goat and swine brucellosis.	Study not conducted in humans.
Ron-Román *et al* 2019	Bayesian evaluation of three serological tests for detecting antibodies against brucella spp. Among humans in the Northwestern Part of Ecuador.	Reference test was not culture.
Hassanain and Ahmed 2012	Efficacy of serological tests in comparison with PCR for diagnosis of brucellosis.	Reference test was not culture.
Zambriski *et al* 2010	Assessment of Brucella melitensis disease burden in lactating goats in Mizque, Bolivia.	Study not conducted in humans.
Garin-Bastuji *et al* 1998	Brucella melitensis infection in sheep: Present and future.	Study not conducted in humans.
Cift *et al* 2018	Comparison of inflammatory markers between brucella and non-brucella epididymo-orchitis.	RBT not evaluated.
Irvem *et al* 2015	Comparison of a New and Rapid Method, Brucella Coombs Gel Test with the Other Methods in the Serological Diagnosis of Brucellosis.	RBT not evaluated.
Pakzad *et al* 2012	Polymerase chain reaction (PCR) diagnosis of human brucellosis (l7/l12 and 16srRNA genes) compared with immunocapture-agglutination test (brucellacapt) and common serological tests.	Reference test was not culture.
Tiwari *et al* 2011	Evaluation of the Recombinant 10-Kilodalton Immunodominant Region of the BP26 Protein of Brucella abortus for Specific Diagnosis of Bovine Brucellosis.	Study not conducted in humans.
Kim *et al* 2010	Real-time PCR Assay Based Glyceraldehydes 3-Phosphate Gene for Identification of Brucella sp.	RBT not evaluated.
Marianelli *et al* 2008	Evaluation of molecular methods for the detection of Brucella species in water buffalo milk.	Study not conducted in humans.
Aliskan 2008	The value of culture and serological methods in the diagnosis of human brucellosis.	Study is narrative review.
Irmak *et al* 2004	Use of the Brucella Igm and IgG flow assays in the serodiagnosis of human brucellosis in an area endemic for brucellosis.	RBT not evaluated.
Clavijo *et al* 2003	Comparison of a dipstick assay for detection of Brucella-specific immunoglobulin M antibodies with other tests for	RBT not evaluated.

	serodiagnosis of human brucellosis.	
Diazaparicio *et al* 1994	Evaluation of serological tests for diagnosis of brucella-melitensis infection of goats.	Study not conducted in humans.
Islam *et al* 2018	Molecular Detection of Brucella spp. from Milk of Seronegative Cows from Some Selected Area in Bangladesh.	Study not conducted in humans.

S2 Table. Search terms used for titles and abstracts and free full text for MEDLINE, EMBASE, WEB OF SCIENCE.

**Search terms**
Rose Bengal test OR Rose Bengal
Brucella OR Brucellosis
Human Brucellosis OR Human
Sensitivity OR Specificity OR Diagnostic performance OR Performance
(Rose Bengal test OR Rose Bengal) AND (*Brucella* OR Brucellosis)
(Rose Bengal test OR Rose Bengal) AND (Human Brucellosis OR Human)
(Rose Bengal test OR Rose Bengal) AND (*Brucella* OR Brucellosis) AND (Human Brucellosis OR Human)
(Rose Bengal test OR Rose Bengal) AND (Human Brucellosis OR Human) AND (Sensitivity OR Specificity OR Diagnostic performance OR Performance)
(Rose Bengal test OR Rose Bengal) AND (*Brucella* OR Brucellosis) AND (Human Brucellosis OR Human) AND (Sensitivity OR Specificity OR Diagnostic performance OR Performance)
